# Examination of Reticulocytosis among Chronically Transfused Children with Sickle Cell Anemia

**DOI:** 10.1371/journal.pone.0153244

**Published:** 2016-04-26

**Authors:** Megha Kaushal, Colleen Byrnes, Zarir Khademian, Natalie Duncan, Naomi L. C. Luban, Jeffery L. Miller, Ross M. Fasano, Emily Riehm Meier

**Affiliations:** 1 Molecular Medicine Branch, National Institute of Diabetes and Digestive and Kidney Diseases, National Institutes of Health, Bethesda, Maryland, United States of America; 2 Center for Cancer and Blood Disorders, Children’s National Health System, Washington, D.C., United States of America; 3 Division of Diagnostic Imaging and Radiology, Children’s National Health System, Washington, D.C., United States of America; 4 Department of Pediatrics, The George Washington University School of Medicine and Health Sciences, Washington, D.C., United States of America; 5 Indiana Hemophilia and Thrombosis Center, Indianapolis, Indiana, United States of America; Emory University/Georgia Insititute of Technology, UNITED STATES

## Abstract

Sickle cell anemia (SCA) is an inherited hemolytic anemia with compensatory reticulocytosis. Recent studies have shown that increased levels of reticulocytosis during infancy are associated with increased hospitalizations for SCA sequelae as well as cerebrovascular pathologies. In this study, absolute reticulocyte counts (ARC) measured prior to transfusion were analysed among a cohort of 29 pediatric SCA patients receiving chronic transfusion therapy (CTT) for primary and secondary stroke prevention. A cross-sectional flow cytometric analysis of the reticulocyte phenotype was also performed. Mean duration of CTT was 3.1 ± 2.6 years. Fifteen subjects with magnetic resonance angiography (MRA) -vasculopathy had significantly higher mean ARC prior to initiating CTT compared to 14 subjects without MRA-vasculopathy (427.6 ± 109.0 K/μl vs. 324.8 ± 109.2 K/μl, *p*<0.05). No significant differences in hemoglobin or percentage sickle hemoglobin (HbS) were noted between the two groups at baseline. Reticulocyte phenotyping further demonstrated that the percentages of circulating immature [CD36(+), CD71(+)] reticulocytes positively correlated with ARC in both groups. During the first year of CTT, neither group had significant reductions in ARC. Among this group of children with SCA, cerebrovasculopathy on MRA at initiation of CTT was associated with increased reticulocytosis, which was not reduced after 12 months of transfusions.

## Introduction

Sickle cell anemia (HbSS, SCA) is a chronic hemolytic anemia that is characterized by ongoing vaso-occlusion and endothelial damage, which results in progressive organ damage. Neurologic injury is one of the earliest SCA sequelae and a characteristic feature found in pediatric SCA patients[1]. Prior to the onset of routine transcranial Doppler (TCD) screening, overt stroke occurred in approximately 11% of SCA patients before 20 years of age[2]. Natural history studies reveal that nearly 70% of SCA patients will suffer a recurrent stroke if left untreated. Additionally, almost 40% of SCA patients who have an overt stroke also have evidence of vasculopathy on magnetic resonance angiography [3], placing this group of patients at the highest risk for recurrent stroke [4]. Moreover, 45% of SCA patients who have had an overt stroke will suffer progressive neurologic damage due to both overt and silent cerebral infarctions, despite chronic transfusion therapy (CTT) [5].

CTT is the current standard of care for SCA patients who have had a stroke or abnormal TCD [6,7]. CTT regimens are designed to: 1) target reduction of HbS to 30% or less [8–10], and 2) increase hemoglobin levels in order to improve the blood’s oxygen carrying capacity[11]. Therefore, with appropriate TCD screening and early implementation of CTT in high-risk patients, the incidence of stroke has decreased substantially [12,13].

SCA erythropoiesis is manifested by lifelong reticulocytosis, which begins during early infancy. In SCA, increased numbers of immature reticulocytes are released into the circulation to help maintain adequate tissue oxygenation. Immature cells are referred to as “stress” or "shift" reticulocytes, classically identified by their lobulated surfaces and aggregation of cytoplasmic reticulin granules [14–17]. More recently, flow cytometry has been used to identify stress reticulocytes according to the expression of surface proteins, including CD71 (transferrin receptor) and CD36 (thrombospondin receptor) [18,19]. Although the clinical significance of the large absolute number of peripheral blood immature reticulocytes in SCA patients is not fully understood, increased reticulocytosis during infancy has been associated with an increased risk of SCA-related hospitalizations [20]. Similarly, high levels of reticulocytosis were associated with an increased risk for cerebrovasculopathy in SCA patients[21]. While CTT in SCA patients generally increases the total hemoglobin[8], the effects upon the compensatory reticulocytosis have not been fully determined. To better understand the link between ongoing reticulocytosis and cerebrovascular disease in chronically transfused SCA patients, we conducted a single center, retrospective study to determine the level of reticulocytosis during the first year of CTT, along with a cross-sectional analysis of the peripheral blood reticulocyte phenotype in CTT patients.

## Materials and Methods

### Subject enrollment

Pediatric SCA patients were recruited from the comprehensive Sickle Cell Program at Children’s National Health System. Approval for the research protocol and consent documents pertaining to this study was granted by the Children’s National Health System and National Institute of Diabetes and Digestive and Kidney Diseases Institutional Review Boards. Written consent was obtained from patients over 18 years of age and written permission was obtained from the parent or legal guardian of patients younger than 18 years of age prior to enrollment. Written assent was obtained from patients ages 7–17 years prior to enrollment. Each patient received a unique alpha-numeric code upon enrollment which was used to anonymize the data collected. Patients between the ages of 2–21 years who had been receiving CTT for at least one year for stroke or abnormal TCD were eligible for this study. CTT patients had no prior treatment with hydroxyurea, nor was it administered concurrently in the transfused subjects. A non-transfused control group (n = 6) of pediatric SCA patients not receiving hydroxyurea was also studied for comparison. Samples were obtained from control patients at steady state (no acute events in the 30 days, no transfusions in the past 60 days). We intentionally selected this control SCA sample to include older patients who did not have a history of an abnormal TCD, overt stroke, and who were not on chronic transfusion therapy, since these patients theoretically have a lower risk of having cerebral vasculopathy. This control sample was included to differentiate the hematologic parameters with our cohort of those patients on chronic transfusions with and without vasculopathy.

This study incorporated a retrospective chart review to determine the hematological effects of transfusion during the first year of CTT. The CTT program at Children’s National requires that peripheral blood samples be drawn 4–72 hours prior to scheduled RBC transfusion to plan for the amount of blood to be transfused. Hematologic data, including absolute reticulocyte count (ARC), were collected and recorded from 3 time points: prior to the start of CTT (baseline T_0_), and later at 6- and 12-months post initiation of CTT (T_6_ and T_12_, respectively). Automated complete blood counts and ARC were measured using a Sysmex XE hematology analyser (Sysmex America, Mundelein, IL). Hemoglobin S levels were quantitated using capillary zone electrophoresis (Sebia, Norcross, GA).

The CTT patients were divided into two groups according to the presence of vasculopathy on magnetic resonance angiography (MRA) at the time of CTT initiation; MRA- group: Abnormal TCD or overt stroke in the absence of MRA detected vasculopathy at the time of initiation of CTT. MRA+ group: Abnormal TCD or overt stroke and vasculopathy detected by MRA at the time of CTT initiation. For each subject, amount of vasculopathy was retrospectively graded by a pediatric neuroradiologist. As shown in Table 1, abnormal cerebral vasculature was graded as mild stenosis (25–50% narrowing), moderate stenosis (50–75% narrowing), or severe stenosis (75–100% narrowing). MRA+ vasculopathy was characterized as the presence of stenosis in the internal carotid artery or the first segments of the anterior, middle, and posterior cerebral arteries. Patients were included in the MRA+ group if vasculopathy was noted at the initiation of CTT. Overt stroke was defined as neurologic findings (lasting more than 24 hours) with new findings of acute cerebral ischemia on head CT or brain MRI. Silent cerebral infarctions were not included in the group stratification.

**Table 1:** 

	MRA Negative	MRA Positive	Control
**N**	14	15	6
**Mean Age (years, ± SD)** ^**^**^	4.1 ± 3.5	6.5 ± 4.3	11.0 ± 3.9
**Male (%)**	5 (36)	12 (80)	3 (50)
**Indication for Transfusion**	TCD^*^: 10	TCD^*^: 9	NA
	Stroke^†^: 4	Stroke^†^: 6	
**Vasculopathy**^**‡**^	NA	ICA: mild (2) mod(4) severe (4)	NA
		ACA: mild (3)^#^ mod (3)^#^ severe (2)^#^	
		MCA: none	

*TCD: abnormal Transcranial Doppler

^†^Stroke: overt stroke; NA: Not applicable; ICA: internal carotid artery; ACA: anterior cerebral artery; MCA: middle cerebral artery.

^‡^Please see text for vasculopathy grading system.

^#^denotes bilateral vasculopathy in 1/3 mild, 2/3 mod, and 1/2 severe.

^^^ denotes mean age at the time of CTT initiation

### Transfusion regimens

CTT patients were prescribed transfusions every 3–4 weeks with the goal of maintaining HbS levels of ≤30%, according to the standard clinical care guidelines of the Children’s National Chronic Transfusion Program. Transfusion modalities included either: 1) simple transfusion (ST) of 10–15 mL/kg RBCs with the post-transfusion Hb goal of 11.5–12.0 g/dL; 2) partial manual exchange transfusion (PME): removal of 5–8 mL/kg of whole blood prior to transfusion of 10–15 mL/kg RBCs with the post-transfusion Hb goal of 11.5–12.0 g/dL; or 3) automated RBC exchange (1) with the post-transfusion goal of 25–30% fraction of cells remaining and Hb approximately 0–2 g/dL above the pre-transfusion Hb. All RBC units were pre-storage leukoreduced, sickle-negative, ABO/Rh compatible, C/c, E/e, and Kell matched with additional antigenic matching dependent on alloantibody identification. The decision of which transfusion modality employed was made by the patient’s primary hematologist based on the patient’s degree of iron overload and baseline hemoglobin.

### Reticulocyte flow cytometry

In addition to data gathered during retrospective chart review, discarded blood was collected immediately prior to a scheduled transfusion from each CTT subject for a cross-sectional flow cytometric analysis of the reticulocyte phenotype after a minimum of 6 months on CTT, referred to as T_x_. One T_x_ sample was drawn 2 months after the subject had started CTT for secondary stroke prevention, but the HbS% was <30% as this subject had an exchange transfusion at the time of the acute stroke. For control patients, blood was collected at steady state as defined by the absence of pRBC transfusion within 60 days, and the absence of SCA-related acute clinical events at least 30 days prior to sample collection. Reticulocyte phenotyping included RNA detection with thiazole orange, as well as the quantitation of immature reticulocytes by detection of transferrin receptor (CD71) and the thrombospondin receptor (CD36) on the reticulocyte membrane. For flow cytometry analysis, blood samples were stored at 4° Celsius and analysed within 72 hours of collection. Cells were stained for CD71 (transferrin, Leinco, St. Louis, MO), glycophorin A (GPA, CD235a, Invitrogen, Carlsbad, CA), CD45 (BD Biosciences, San Jose, CA), CD36 (thrombospondin, Beckman Coulter, La Brea, CA) and thiazole orange (Sigma Aldrich, St. Louis, MO) and quantitated using a BD FACS Aria I flow cytometer (Becton Dickinson, San Jose, CA). The reticulocyte population was defined as the GPA positive, CD45 negative cells with positive thiazole orange fluorescence. At least 5,000 cells were recorded after doublet discriminator gating. Cells with fluorescence of more than two standard deviations above the unstained controls were defined as positive.

### Statistical analyses

Data analyses were performed using Microsoft Excel 2013 and IBM SPSS Statistics 23.0 (IBM, Armonk, NY). Means were calculated with standard deviation. Two-tailed Student’s *t-*tests were performed to compare means. Medians were calculated with interquartile ranges. Two-tailed Mann-Whitney U-tests were used to compare medians. Correlations were evaluated using two-tailed Pearson product-moment correlation coefficients. A *p*-value of <0.05 was considered statistically significant.

## Results

### Clinical and hematologic phenotype of the cohort

Thirty-five SCA patients were enrolled in this study. Of these, 29 were receiving CTT and 6 were in the non-transfused control group of children with SCA not receiving hydroxyurea. The CTT group had 17 males and 12 females. The CTT group was significantly younger than the control group (5.3 ± 4.1 years vs. 11.0 ± 3.9 years, respectively, *p*<0.05). The mean duration of CTT was 3.1 ± 2.6 years (range: 0.2 to 10.5 years). Fourteen patients were MRA- and 15 patients had vasculopathy on MRA (MRA+) at CTT initiation. The mean age for MRA- and MRA+ was 4.1 ± 3.5 years and 6.5 ± 4.3 years (*p* = 0.10), respectively, at baseline. For those subjects in the MRA+ group, 7 subjects had ICA vasculopathy, 6 subjects had ACA vasculopathy, and 2 had both ICA and ACA vasculopathy (Table 1).

**Fig 1:**
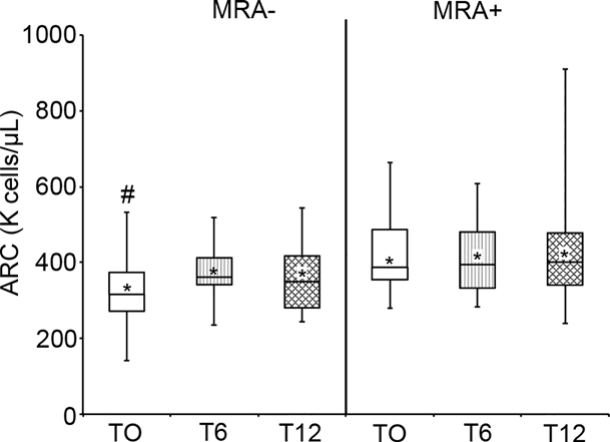
Pre-transfusion ARC during initial year of CTT. Mean pre-transfusion values for absolute reticulocyte counts [ARC (K cells/μL)] are shown at T_0,_ T_6,_ or T_12_ for MRA- and MRA+ groups by the asterisk (*) in the box and whisker plot. Median [Interquartile Range] for both groups are illustrated by the horizontal lines within the box (MRA-: T_0_: 316.2 K/μl [271.4, 373.5]; T_6_: 361.2 K/μl [341.4, 412.2]; T_12_: 349.4K/μl [280.3, 417.2]; MRA+: T_0_: 387.6 K/μl [354.2, 486.5]; T_6_: 394.3 K/μl [332.6, 480.3]; T_12_: 400.8 K/μl [340.6, 478.0]). # denotes a statistically significant difference between mean (and median) T_0_ values for MRA+ and MRA- groups. There was no statistical significance between the means or medians of MRA+/MRA- T_6_ and T_12_ ARC values.

There was no significant difference in the ARC values measured at the start of CTT (T_0_) compared to T_6_ or T_12_ months post-CTT initiation within either the MRA- or MRA+ group (MRA-: T_0_: 324.8 ± 109.2 K/μl; T_6_: 375.4 ± 75.8 K/μl; T_12_: 355.8 ± 90.8 K/μl; *p* = 0.17, *p* = 0.43, respectively; MRA+:T_0_: 427.6 ± 109.0 K/μl; T_6_: 417.3 ± 105.1 K/μl; T_12_: 429.3 ± 168.3 K/μl; *p* = 0.80, *p* = 0.97, respectively). However, the T_0_ ARC differed significantly between the two groups [MRA-: 324.8 ± 109.2 K/μl vs. MRA+: 427.6 ± 109.0 K/μl; *p*<0.05] (Fig 1). The hemoglobin within the MRA- group and MRA+ group increased after initiation of CTT as expected. The percent of HbS within the MRA- group was significantly lower at 6 and 12 months post-CTT compared to initiation (T_0_: 82.0 ± 5.7% vs. T_6_: 38.5 ± 7.9%, *p*<0.001; vs. T_12_: 34.3 ± 11.1%, *p*<0.001). The HbS% within the MRA+ group was similarly decreased (T_0_: 87.4 ± 7.1% vs. T_6_: 36.8 ± 10.1%, *p*<0.001; vs. T_12_: 36.7 ± 13.4%, *p*<0.001). No significant differences in hemoglobin or HbS levels were present between the two groups when the same time points were compared. Twenty-seven of 29 CTT patients (93.1%) received ST and 2 patients (6.9%) received PME at the initiation of transfusion (T_0_). There was no significant difference in ARC between the two CTT modalities at T_0,_ T_6,_ or T_12_ (ST Mean ARC T_0:_ 382.8 ± 119.9 K/μl; T_6_: 402.3 ± 94.1 K/μl; T_12_: 401.5.8 ± 145.4 K/μl; PME Mean ARC T_0:_ 290.7 ± 91.0 K/μl; T_6_: 326.7 ± 35.0 K/μl; T_12_: 342.1 ± 90.2 K/μl; *p* values: T_0:_ *p* = 0.30; T_6_: *p* = 0.27; T_12_: *p* = 0.50).One patient who was changed from simple to PME at T_12_ did not have a significant difference in ARC.

### Cross-sectional reticulocyte analysis

**Fig 2:**
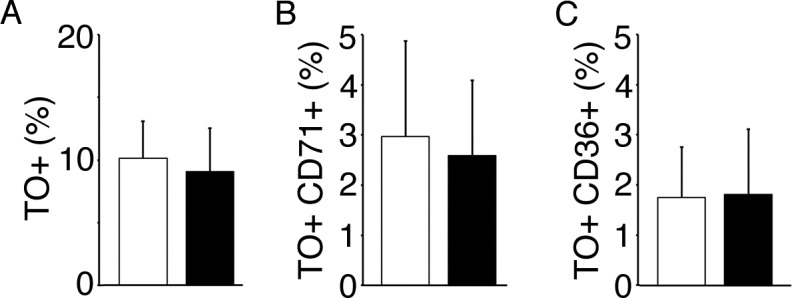
Reticulocyte phenotyping in CTT and non-transfused control groups. Mean TO%, mean CD71+, and mean CD36+ (A,B,C) of non-transfused control group (open bars) and transfused group (black bars). Standard deviation bars are shown.

**Fig 3:**
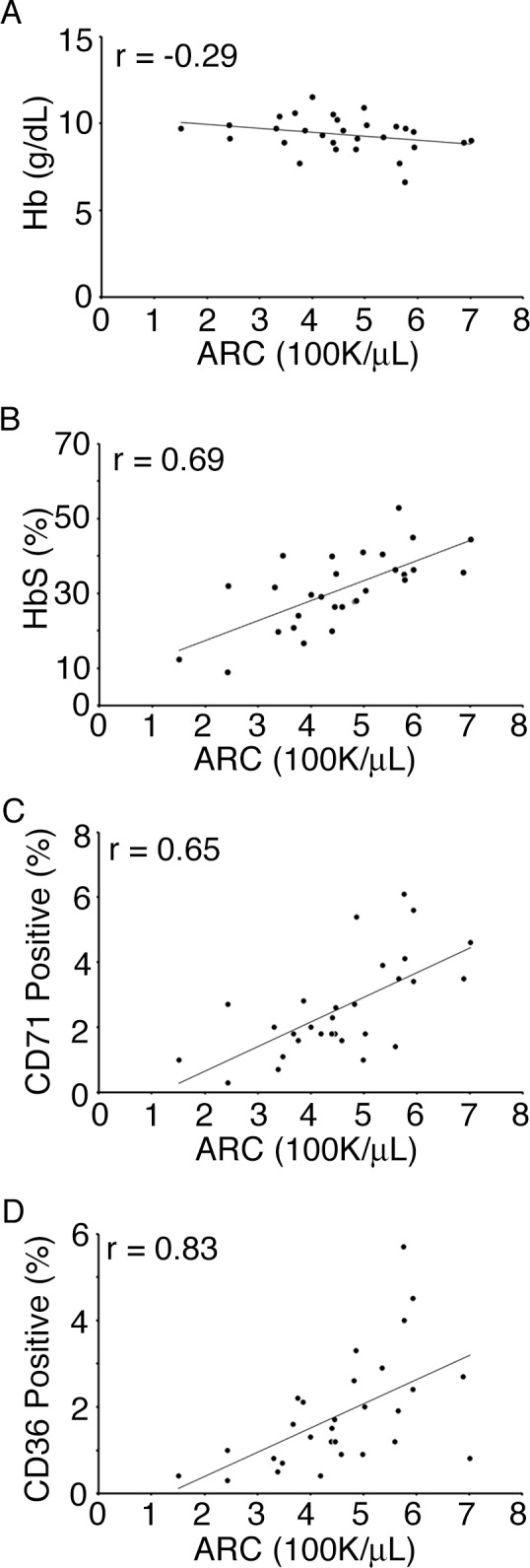
Analysis of ARC correlations. Values for A) hemoglobin [Hb (g/dL)], B) percentage of sickle hemoglobin [HbS (%)], C) percentage of reticulocytes with detected plasma membrane transferrin [CD71 (%)], and D) percentage of reticulocytes with detected plasma membrane thrombospondin receptor [CD36 (%)] are shown on the y-axis and plotted with the correlated absolute reticulocyte counts [ARC (100K/μL)] on the x-axis of each panel. Linear trend lines and correlation coefficients (r) are shown.

Equivalent percentages of circulating reticulocytes (TO+) were measured in the CTT and non-transfused control groups (Fig 2A). The mean TO+ erythroid fraction for the CTT group was 9.2 ± 3.5% compared to 10.0 ± 3.3% from the non-transfused controls. The proportion of CD71+ cells did not decrease with CTT (2.6 ± 1.5% vs. 3.0 ± 1.9%, CTT vs. control, respectively, *p* = 0.41, Fig 2B). The mean CD36+ population also did not decrease with CTT (1.8 ± 1.3% vs.1.9 ± 1.0%, CTT vs. controls, respectively, *p* = 0.91, Fig 2C). The CBC and ARC were measured using samples collected prior to a scheduled transfusion which were also used for flow cytometry. Hemoglobin levels and ARC did not correlate in this group of chronically transfused patients. (r = -0.29, *p* = 0.13, Fig 3A). However, the ARCs were correlated with HbS levels in the CTT group (r = 0.69, *p*<0.01, Fig 3B). In addition, an increased proportion of circulating immature reticulocytes were associated with increased ARC [CD71+ and CD36+ populations; r = 0.65 (*p*<0.01) and 0.83 (*p*<0.01), respectively, Fig 3C and 3D].

## Discussion

Over the last two decades, chronic transfusions have become the standard therapy for reduction or prevention of cerebrovascular sequelae in pediatric SCA[22]. Mixing studies of HbSS and HbAA blood [23] provided a scientific basis for initial clinical studies of chronic transfusions by reducing blood viscosity as well as the portion of HbS-containing erythrocytes in circulation [8,24]. In general, CTT regimens are aimed toward reducing the HbS percentage to below thirty percent while maintaining total post-transfusion hemoglobin under 12 g/dL [6,12]. Target HbS levels ranging from less than 20% in high-risk patients with progressive vasculopathy, and HbS levels of up to 50% in patients who have had stable disease for greater than 5 years on CTT have also been utilized for primary and secondary stroke prevention [25,26].

Current data suggests that ARC may be a useful SCA severity marker. Based upon the recent use of ARC as a predictive marker for SCA pathologies (including cerebrovascular disease)[20,21,27], we hypothesized that higher ARC levels among CTT patients may predict more severe cerebrovascular disease. Even among this small cohort of patients, baseline ARC levels (T_0_) were significantly higher among the SCA patients who had vasculopathy detectable by MRA at the initiation of CTT. The degree of reticulocytosis among this group was striking with an average level of more than 400 K/μl, and not significantly different from the non-transfused control SCA cohort. No difference in the ARC was seen when transfusion modalities or pre-transfusion hemoglobin levels were compared. Larger, prospective studies are needed to confirm these results.

Consistent with previous reports [26], lower HbS levels were correlated with lower ARC (Fig 3). However, when compared to baseline ARC levels measured prior to the initiation of transfusion therapy (T_0_), we found that CTT did not result in a significant reduction in ARC levels measured immediately prior to scheduled transfusions after 6 and 12 months of CTT. The lack of suppression of reticulocytosis despite a rise in hemoglobin remains unexplained and intriguing. Similarly high levels of transfusion-related ARC have not been reported among chronically transfused thalassemia patients[26,28,29], perhaps due to differences in the degree of ineffective erythropoiesis or other distinct features of these hemoglobinopathies. Reticulocyte levels decrease during the first two weeks following pRBC transfusion in SCA patients, suggesting that erythropoiesis is initially suppressed [30,31]. However, the high levels of reticulocytosis 3–4 weeks after transfusion that we observe imply a significant rebound in erythropoietic activity in pediatric SCA patients just prior to the subsequent scheduled transfusion. The positive correlation between ARC levels and the proportion of immature reticulocytes in the peripheral circulation that we report here suggests that the reticulocyte may play an important role in SCA pathophysiology since patients with vasculopathy on MRA had higher ARC levels at CTT initiation and immature reticulocytes have a higher number of adhesion markers on their surface (Fig 3).

HbS-containing erythrocyte adherence to the endothelium is a key factor in the pathophysiology of SCA. When sickle erythrocytes with low HbF levels become dehydrated or deoxygenated, the HbS molecules polymerize, resulting in the characteristic shape change of the erythrocyte and exposure of phosphatidylserine (PS) on the cell surface. It is known that PS exposure causes red cell adherence to the vascular endothelium [32,33]. Additionally, thrombospondin and laminin adhere to sickle erythrocytes and reticulocytes via the thrombospondin receptor (CD36) and coagulation factors are activated as evidenced by increased fibrin and tissue factor levels in SCA patients[34,35]. Clustering of CD36+ reticulocytes also strengthen the interaction of the red blood cell and endothelium [36,37]. Examination of the reticulocyte profiles of age-matched healthy non-SCA controls revealed absence of CD36 expression in the peripheral blood erythroid cells (see S1 Fig) which is consistent with previous reports [36]. Additionally, patients with SCA have nearly ten times the amount of CD36+ expression on their erythrocytes compared to patients with other hemolytic anemias that are not associated with vasculopathy [38]. Hence, we postulate that the presence of CD36 on the surface of immature sickle reticulocytes may contribute to the pathophysiology seen in SCA-associated vasculopathy, based on the associations observed between the higher ARC in patients with MRA-documented cerebrovasculopathy prior to initiating CTT which was unchanged after a year of CTT. This data suggest that this association is intrinsic to the patient regardless of CTT, and supports our previous data demonstrating that reticulocytosis is a hematologic marker of serious disease complications [39]. More detailed studies of reticulocyte adhesion properties in addition to the kinetics of their production post-transfusion are needed to better understand the biological relevance of ongoing or post-transfusional rebound reticulocytosis in these children. Additionally, efforts should continue to identify the signalling processes and cascade of events leading to reticulocyte adhesion.

Importantly, targeted reduction of HbS to 30% significantly reduces but does not eliminate recurrent cerebral infarcts in children with SCA [40]. While the goal of CTT for thalassemia major patients is to suppress erythropoiesis, the frequency of and amount of transfusion in SCA patients are titrated based on HbS and hemoglobin levels. In our cohort, HbS levels were slightly higher than the target HbS of less than 30%, which is similar to the HbS average levels (34%) reported for patients enrolled in the Stroke with Transfusion Changing to Hydroxyurea (SWiTCH) study [41,42]. Hydroxyurea combined with phlebotomy was recently reported as an acceptable alternative for patients with abnormal TCD and minimal vasculopathy versus the current treatment approach of transfusion combined with chelation therapy [43]. While the mean HbS level in the transfusion arm of the TCD with Transfusion Changing to Hydroxyurea (TWiTCH) trial was 28%, mean HbS level in the hydroxyurea/phlebotomy group was significantly higher (71%) at study completion. Reticulocytosis persisted in the patients who continued transfusions at similar levels to our current report (mean ARC: 329 ± 112 K/μl), while patients randomized to hydroxyurea and phlebotomy had a significantly lower mean ARC (181 ± 86 K/μl) as well as lower white blood cell, absolute neutrophil, and platelet counts. No patients in either group had a new cerebral infarct and the only patient with progressive vasculopathy was in the transfusion/chelation arm of the study, which suggests that the lowering of these hematologic values closer to the normal range, including the reduction of ARC, by hydroxyurea may play a key role in stroke prevention in this cohort of patients. The sickle reticulocyte may be significant in the SCA pathophysiology and an important therapeutic target. Ultimately, further studies are needed to identify and treat SCA patients who are destined to develop progressive neurological disease while receiving chronic transfusions. Those investigations should include identifying the role of ongoing reticulocytosis on progressive vasculopathy in this population.

## Supporting Information

S1 Fig Patient Specific Reticulocyte Phenotyping. Representative grey-scale, contour plot flow cytometric analyses of reticulocytes from a non-transfused SCA patient (A,B,C), a CTT patient (D,E,F) and healthy non SCA control (G,H,I) are shown. Reticulocytes were identified with thiazole orange (TO) staining (A,D,G), while reticulocyte maturity was quantified using antibodies directed towards CD71 (B,E,H) and CD36 (C,F,I). Horizontal and vertical lines in each denote separation of negative and positive fluorescence levels (see Methods). Absence of CD36 expression is shown in healthy non SCA control.
(TIF)

## Abbreviations

ARC: absolute reticulocyte count
CTT: chronic transfusion therapy
MRA: magnetic resonance angiography
SCA: sickle cell anemia
HbS: sickle hemoglobin
CD36: thrombospondin receptor
TCD: transcranial Doppler
CD71: transferrin receptor
ST: simple transfusion
